# Unrepaired persistent truncus arteriosus in a 38-year-old woman with an uneventful pregnancy

**DOI:** 10.5830/CVJA-2015-005

**Published:** 2015

**Authors:** Dorra Abid, Sahar Ben Kahla, Souad Mallek, Leila Abid, Samir Kammoun, Emna Daoud, Hela Fourati, Zeineb Mnif

**Affiliations:** Cardiology Department, Hedi Chaker Hospital, Sfax, Tunisia; Cardiology Department, Hedi Chaker Hospital, Sfax, Tunisia; Cardiology Department, Hedi Chaker Hospital, Sfax, Tunisia; Cardiology Department, Hedi Chaker Hospital, Sfax, Tunisia; Cardiology Department, Hedi Chaker Hospital, Sfax, Tunisia; Department of Radiology, Hedi Chaker Hospital, Sfax, Tunisia; Department of Radiology, Hedi Chaker Hospital, Sfax, Tunisia; Department of Radiology, Hedi Chaker Hospital, Sfax, Tunisia

**Keywords:** persistent truncus arteriosus, adult, echocardiography, pulmonary artery hypertension, magnetic resonance imaging

## Abstract

Persistent truncus arteriosus (PTA) is a rare conotruncal defect, defined as a single arterial vessel arising from the heart, which gives origin to the systemic, pulmonary and coronary circulations. It has an extremely poor prognosis and carries a high mortality rate during the early years of life unless surgically repaired. A few known cases have been reported of patients reaching maturity, and exceptionally, patients suffering from this disease having lived into the fourth decade.

The purpose of this report was to present a new case of PTA type 1, diagnosed by echocardiography and MRI, in a 41-year-old woman, with the peculiarity of long survival into adult life. She had also experienced a full-term pregnancy and delivery of a normal infant three years prior to her diagnosis. Pulmonary vascular disease made her condition inoperable but she was doing well with medical management after a follow up of 15 months. Based on this work, we concluded that pulmonary arterial hypertension is deleterious for life in some cardiovascular diseases, but in others, allows survival, as occurred in these patients with PTA. The patient’s clinical course and anatomical findings are reported, along with factors that may have contributed to her longevity.

## Abstract

Persistent truncus arteriosus (PTA) is an uncommon congenital heart disease (CHD) that was first described by Wilson in 1798. In 1976, Calder *et al*. reported it accounted for approximately 0.7 to 1.2% of all congenital heart malformations and occurred equally in men and women.^1^

Truncus arteriosus (TA) is defined as a single arterial vessel, usually arising from both the left and the right ventricle, which gives rise to the systemic, pulmonary and coronary artery circulations.^1^ It is mostly associated with a large, non-restrictive ventricular septal defect (VSD) situated below the semilunar truncal valve.

PTA is also reported to be associated with a high rate of mortality if uncorrected. Calder *et al*. stated that about 65% of patients treated medically did not survive beyond six months of age, and more than 90% who did not have surgical repair died before one year of age.^1^ Surgical intervention is required to avoid pulmonary vascular disease, which is common in the unrepaired patient. Because of this extremely poor prognosis, PTA is uncommonly encountered in adult life.

Here we report on the unusual case of a woman with unrepaired TA who was evaluated by echocardiography and magnetic resonance imaging (MRI).

## Case report

A 41-year-old housewife initially presented to the Cardiology Department of Hedi Chaker Hospital in Tunisia in November 2011 with symptoms of exercise intolerance and occasional palpitations of several months’ duration. She had no family history of congenital defects. Three years earlier, when she was 38 years old, she gave birth to a normal baby after undergoing an uneventful full-term pregnancy and delivery.

Cyanosis and dyspnoea on exertion had been present throughout her life, but despite this, she appeared to have had a fairly normal life, being able to tolerate daily activities well. Two weeks prior to admission, she reported having experienced an exacerbation of dyspnoea.

She was a small-built woman and appeared deeply cyanosed on examination but not dyspnoeic at rest. Prominent clubbing of the fingers was noticeable. Congestion of the jugular veins was also striking. Her oxygen saturation in room air was about 80%. Physical examination revealed a mild systolic murmur over the left parasternal border and a loud second heart sound in the right second intercostal space.

A 12-lead resting electrocardiogram revealed sinus rhythm, right bundle branch block and high QRS voltage suggestive of biventricular hypertrophy. A chest radiograph revealed marked cardiomegaly with a prominent main pulmonary trunk and increased pulmonary vascularity.

**Figure 1.**
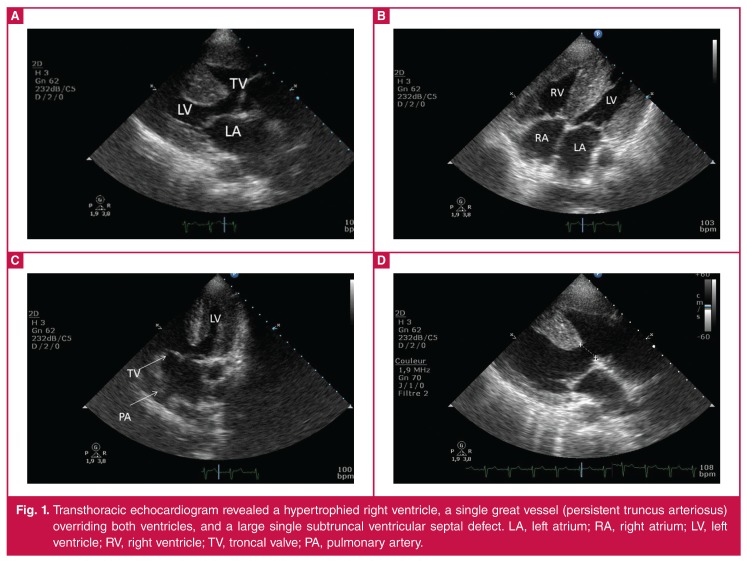
Transthoracic echocardiogram revealed a hypertrophied right ventricle, a single great vessel (persistent truncus arteriosus) overriding both ventricles, and a large single subtruncal ventricular septal defect. LA, left atrium; RA, right atrium; LV, left ventricle; RV, right ventricle; TV, troncal valve; PA, pulmonary artery.

Transthoracic echocardiography indicated a levocardia heart with atrial situs solitus and concordant atrioventricular connections. Marked biventricular hypertrophy in the fourchamber view was also evident. The left ventricle demonstrated a normal ejection fraction. The most striking finding was a single large vessel arising from the base of the heart, with mild regurgitation related predominantly to the summit of the right ventricle (70%). A large, non-restrictive outlet VSD was noted beneath the truncal valve (Fig. 1). Neither the pulmonary artery (PA) nor the pulmonary valve could be seen.

**Figure 2.**
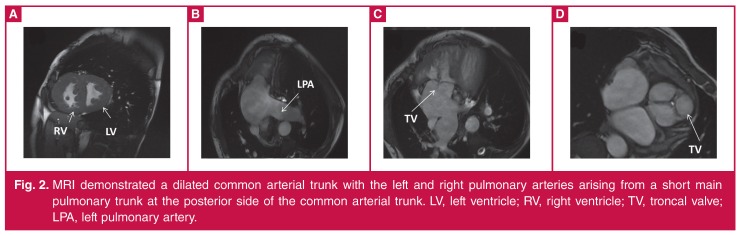
MRI demonstrated a dilated common arterial trunk with the left and right pulmonary arteries arising from a short main pulmonary trunk at the posterior side of the common arterial trunk. LV, left ventricle; RV, right ventricle; TV, troncal valve; LPA, left pulmonary artery.

Cardiac MRI was also performed to better delineate the origin of the pulmonary arteries. It demonstrated a dilated common arterial trunk with the left and right pulmonary arteries arising from a short main pulmonary trunk at the posterior side of the common arterial trunk. The left ventricle (LV) was normal in size. The right ventricle (RV) was also normal in size with concentric hypertrophy. A large, subarterial VSD was noted beneath the truncal valve, which was trileaflet, with mild insufficiency (Fig. 2).

From on the above findings, for this patient, a diagnosis in keeping with a conotruncal anomaly could best be classified as type 1, based on Collette and Edwards’ classification.^2^ Furthermore, the thoracic vasculature was significantly altered, with irreversible pulmonary hypertension. Taking this into account, conservative management was recommended.

The patient appeared to make good progress on medical treatment, which included bed rest and fluid restriction. She was advised against pregnancy, considering her mature age and in light of the underlying severe pulmonary hypertension. Her condition appeared to have remained stable 15 months after discharge from hospital. Chromosomal studies were not undertaken, however her child appeared in a good state of health.

## Discussion

PTA usually includes a large VSD with the presence of a significant left-to-right shunt, and is dependent on the resistance ratios between the systemic and pulmonary circulation. Indeed, pulmonary vascular resistance (PVR) decreases during the first weeks of life, and neonates experience congestive heart failure because of the increased pulmonary blood flow, unless the pulmonary arteries are hypoplastic or stenosed or there is persistently elevated PVR. These factors may delay the appearance of symptoms and babies appear mildly cyanosed due to the high PVR.

According to Marcelletti *et al.*,^3^ these first effects are mainly beneficial and some patients unusually survived through to adulthood. Such survival is achieved only at the price of subjecting the pulmonary vasculature to the effects of severe pulmonary hypertension due to occlusive intimal fibro-elastosis.^3^

Echocardiography is a reliable, non-invasive, first-line imaging tool that proved to be beneficial in the diagnosis of differentiating this lesion from pulmonary atresia with VSD. The hallmark of PTA is that only a single semilunar valve is seen.^4^

MRI, although expensive and not easily accessible to all patients, is a complementary modality that has been shown to be accurate in the diagnosis and follow up of CHD.^5^ It is a useful adjunct, currently recognised by paediatric cardiologists and cardiac surgeons because it includes a wide field of view and multiplanar capabilities and reconstructions. The strength of MRI includes comprehensive access and coverage, providing imaging of all parts of the right ventricle, pulmonary arteries, pulmonary veins and aorta.^6^

Our case illustrates the major role of cardiac MRI, due to it being a fairly safe technique that allows precise definition and high resolution, and in this case, it demonstrated the complex anatomy of PTA. In this patient, the truncal valve was trileaflet and competent, which may have played an important role in her survival, since it is well established that truncal valve insufficiency is associated with higher rates of early and late mortality.^7^

Eisenmenger physiology is an absolute contraindication to pregnancy. Maternal mortality is reported to be as high as 36%.^8^ However, in this case, the patient had not received prior counselling before she fell pregnant, since her diagnosis was made three years after delivery. She was fortunate not to have had a complicated pregnancy.

## Conclusion

Echocardiography and MRI played an important role in detecting the PTA in this patient, which assisted in appropriate management. PTA is an uncommon cardiovascular anomaly with a poor prognosis, and without surgical repair, is regarded to be incompatible with life. This unique case study (type 1 PTA) offers an example of the natural history of an unrepaired complex congenital cardiac disease that overcame the odds of a short life expectancy.
